# Immune network dysregulation associated with child neurodevelopmental delay: modulatory role of prenatal alcohol exposure

**DOI:** 10.1186/s12974-020-1717-8

**Published:** 2020-01-28

**Authors:** Tamara S. Bodnar, Charlis Raineki, Wladimir Wertelecki, Lyubov Yevtushok, Larisa Plotka, Irina Granovska, Natalya Zymak-Zakutnya, Alla Pashtepa, Alan Wells, Gordon Honerkamp-Smith, Claire D. Coles, Julie A. Kable, Christina D. Chambers, Joanne Weinberg, and the CIFASD

**Affiliations:** 1 Department of Cellular and Physiological Sciences, University of British Columbia, 3307 – 2350 Health Sciences Mall, Vancouver, BC V6T 1Z3 Canada; 2 Department of Pediatrics, University of California San Diego, La Jolla, USA; 3 OMNI-Net for Children International Charitable Fund, Rivne Oblast Medical Diagnostic Center, Rivne, Ukraine; 4 OMNI-Net for Children International Charitable Fund, Khmelnytsky Perinatal Center, Khmelnytsky, Ukraine; 5 Department of Psychiatry and Behavioral Sciences; Department of Pediatrics, Emory University School of Medicine, Atlanta, USA; 6 Department of Family Medicine and Public Health, University of California San Diego, La Jolla, CA USA

**Keywords:** Cytokines, Immune networks, Fetal alcohol spectrum disorders, Development

## Abstract

**Background:**

Evidence suggests that cytokine imbalances may be at the root of deficits that occur in numerous neurodevelopmental disorders, including schizophrenia and autism spectrum disorder. Notably, while clinical studies have demonstrated maternal cytokine imbalances with alcohol consumption during pregnancy—and data from animal models have identified immune disturbances in alcohol-exposed offspring—to date, immune alterations in alcohol-exposed children have not been explored. Thus, here we hypothesized that perturbations in the immune environment as a result of prenatal alcohol exposure will program the developing immune system, and result in immune dysfunction into childhood. Due to the important role of cytokines in brain development/function, we further hypothesized that child immune profiles might be associated with their neurodevelopmental status.

**Methods:**

As part of a longitudinal study in Ukraine, children of mothers reporting low/no alcohol consumption or moderate-to-heavy alcohol consumption during pregnancy were enrolled in the study and received neurodevelopmental assessments. Group stratification was based on maternal alcohol consumption and child neurodevelopmental status resulting in the following groups: A/TD, alcohol-consuming mother, typically developing child; A/ND, alcohol-consuming mother, neurodevelopmental delay in the child; C/TD, control mother (low/no alcohol consumption), typically development child; and C/ND, control mother, neurodevelopmental delay in the child. Forty cytokines/chemokines were measured in plasma and data were analyzed using regression and constrained principle component analysis.

**Results:**

Analyses revealed differential cytokine network activity associated with both prenatal alcohol exposure and neurodevelopmental status. Specifically, alcohol-exposed children showed activation of a cytokine network including eotaxin-3, eotaxin, and bFGF, irrespective of neurodevelopmental status. However, another cytokine network was differentially activated based on neurodevelopmental outcome: A/TD showed activation of MIP-1β, MDC, and MCP-4, and inhibition of CRP and PlGF, with opposing pattern of activation/inhibition detected in the A/ND group. By contrast, in the absence of alcohol-exposure, activation of a network including IL-2, TNF-β, IL-10, and IL-15 was associated with neurodevelopmental delay.

**Conclusions:**

Taken together, this comprehensive assessment of immune markers allowed for the identification of unique immune milieus that are associated with alcohol exposure as well as both alcohol-related and alcohol-independent neurodevelopmental delay. These findings are a critical step towards establishing unique immune biomarkers for alcohol-related and alcohol-independent neurodevelopmental delay.

## Background

Immune system dysregulation has been recognized as a core feature in a wide range of neurodevelopmental disorders including schizophrenia [1, 2], autism spectrum disorder (ASD) [3], cerebral palsy (CP) [4], and others [5]. Notably, accumulating evidence suggests that cytokine imbalances, both peripherally and centrally, may be at the root of many brain-related changes that occur in neurodevelopmental disorders [6] due to the key role of cytokines in brain development and function [7]. From a mechanistic perspective, evidence points to maternal immune activation, induced through a variety of exposures or insults, as an underlying factor in neurodevelopmental disturbances in the offspring (reviewed in [6]); indeed, maternal immune disturbances have been observed in ASD [8, 9] and schizophrenia [10, 11]. In parallel, we have recently shown that alcohol consumption during pregnancy is associated with alterations in the maternal cytokine balance [12]. Notably, maternal immune profiles could also be linked to child neurodevelopmental status, with differential activation/inhibition of cytokine networks in the context of alcohol-related neurodevelopmental delay compared to typical development. Thus, alcohol consumption during pregnancy can be considered an environmental exposure or insult that results in maternal immune dysregulation, with long-lasting consequences for offspring immune function and brain development

Fetal alcohol spectrum disorder (FASD), which occurs as a result of prenatal alcohol exposure, can have a multitude of effects on the developing fetus, ranging from craniofacial abnormalities [13] to dysregulation of physiological systems [14, 15], as well as behavioral [16] and cognitive dysfunction [17]. FASD is arguably the most common neurodevelopmental disorder, with current US prevalence estimated at 1.1 to 5.0% [18], compared to 1.4% for ASD [19]; however, FASD has received very little research attention—particularly in the context of immune function—making evidence for immune dysfunction limited (reviewed in [20]). The first study in this area, a small case study of children with fetal alcohol syndrome (FAS; at the most severe end of the FASD spectrum), showed that children with FAS had increased rates of major and minor infections compared to controls [21], and this increased infection risk associated with prenatal alcohol exposure has been replicated in other studies as well [22–24]. In addition, impairments in cell-mediated and humoral immunity [21] and increased risk of dermatitis [25], eczema [26], and certain cancers [27] have also been reported (reviewed in [28, 29]). All of these immune-related changes may be a result of alcohol-induced programming of the immune system, altering the production and/or release of immune mediators. However, only a single study has investigated this possibility, with results indicating that indeed, chronic alcohol exposure induces immune system disturbances, with elevation of cytokines, including TNF-α, IL-1β, and IL-6, in fetal (cord) blood at birth [30]. Nevertheless, a question that remains is whether these alcohol-induced changes in fetal cytokine levels are enduring and can still be detected later in the postnatal period when alcohol is no longer present. Support for this comes from data demonstrating that immune disturbances are a common lifelong feature of many neurodevelopmental disorders. In ASD, immune disturbances have been detected from childhood [31, 32] through to adulthood [33] (reviewed in [34]). Similarly, in schizophrenia, cytokine changes are being considered as potential biological markers, as differential profiles have been detected across disease states (reviewed in [35]).

One especially challenging feature in characterizing many neurodevelopmental disorders is their heterogeneous nature, with deficits existing along a continuum. As such, specific biomarkers able to differentiate subpopulations most at risk from those showing resilience would be particularly beneficial. Due to the previous findings of immune dysfunction in neurodevelopmental disorders and the important role of the immune system in brain development, immune-based biomarkers may be especially well-positioned to predict the developmental trajectory of these disorders. In fact, the predictive potential of immune-based biomarkers, specifically cytokines, have been examined in ASD, with elevated IL-4 at birth associated with an increased risk of severe ASD, and the finding of a negative association between IL-4 and nonverbal cognitive ability [36]. Nevertheless, immune-based biomarkers have not been investigated in children with prenatal alcohol exposure.

In the current study, we hypothesized that perturbations in the immune environment as a result of prenatal alcohol exposure would program the developing immune system and result in immune dysfunction into childhood. We explored whether different cytokine signatures could be identified in alcohol-exposed children experiencing neurodevelopmental delay compared to those with typical neurodevelopment, with the goal of identifying those children most at risk following prenatal alcohol exposure. Importantly, in order for immune-based biomarkers to be useful beyond being predictive of outcomes, they also need to be disorder-specific. To begin to address the issue of specificity, the incorporation of an alcohol-independent neurodevelopmental delay group allowed for cytokine signatures in this group to be compared to those children with alcohol-dependent neurodevelopmental delay. Another strength of the present study is our adoption of a data analysis strategy focused on the evaluation of networks of interacting cytokines in addition to the evaluation of individual cytokines. To date, over 300 cytokines have been identified and have been shown to affect a myriad of biological processes ranging from early development to degenerative aging processes (reviewed in [37]). Cytokine signaling is complex in that cytokines are redundant and pleiotropic, act in concert with one another, and can be produced by and affect virtually every cell in the body (reviewed in [37, 38]). As a result, it can be difficult to generalize about the impact of any one individual cytokine. Recent major advances in characterizing cytokine networks [39] over the profiling of a small subset of individual cytokines allowed us to take into account this complexity in cytokine function and, we believe, to generate a more representative and functional understanding of the immune environment to which the fetus is exposed.

## Methods

### Cohort description

Pregnant women were enrolled from 2007 to 2012 as part of a longitudinal study conducted at two prenatal care facilities in Western Ukraine (Rivne and Khmelnytsky oblasts) affiliated with the OMNI-Net Ukraine Birth Defects Prevention Program; this longitudinal study is part of the Collaborative Initiative on Fetal Alcohol Spectrum Disorders (CIFASD.org). A detailed description of cohort recruitment methods has been published previously [40–42]. Briefly, women coming in for routine prenatal visits provided demographic and pregnancy history information and were screened using standard questions on alcohol consumption and other exposures. For women reporting drinking at any point during their lifetime, additional questions were included in the interview relating to the quantity and frequency of alcohol consumption according to a previously validated questionnaire used to identify risky drinking during pregnancy [43]. Women were eligible for enrollment in the alcohol-consuming group if they reported moderate to heavy alcohol consumption in the periconceptional period, defined as at least weekly binge-drinking episodes (5+ drinks), or at least 5 occurrences of 3–4 standard drinks, or at least 10 occurrences of 1–2 standard drinks, either in the month of conception or in the most recent month of pregnancy [40]. Women were enrolled in the low/no alcohol consumption contrast group if they met the screening criteria of no binge episodes, minimal or no alcohol in the month around conception, and no drinking in the most recent month of pregnancy [43]. All women were offered information regarding the risks of alcohol consumption during pregnancy. Additionally, all women completed a brief health questionnaire that assessed their current health status, with data collected including rates of high blood pressure and diabetes, current treatment for tuberculosis, viral or autoimmune hepatitis, and chronic liver or pancreatic disease. From the individuals included in the current study, only one mother had high blood pressure and another had hepatitis. All participants provided informed consent, and study protocols were approved by Institutional Review Boards at the Lviv National Medical University, the University of California San Diego, and the University of British Columbia.

### Child outcomes

After delivery, medical records were provided for the infants and the following information was collected: child sex, gestational age, body weight and length, and head circumference. At 6 and 12 months of age, neurodevelopment was assessed via the Bayley Scales of Infant Development Second Edition (BSID-II) [44]. Specifically, the Mental Development Index (MDI), which assesses early cognitive and language development, and the Psychomotor Development Index (PDI), which evaluates body control and manipulation skills were utilized. All scores were standardized by age and corrected for prematurity (< 37 weeks). Children also received a dysmorphology examination to evaluate the physical features of FASD at birth and at 6 and 12 months as per [45].

### Selection of the sample

For the current analysis, children were selected from the overall cohort if they received at least one neurodevelopmental assessment at 6 or 12 months and provided a blood sample at 2–3.5 years of age. Of note, a blood sample was only collected from healthy children (no signs of ongoing infection). Stratification of the sample was based on both maternal alcohol consumption during pregnancy (moderate to heavy alcohol consumption or low to no alcohol consumption) and child outcomes on the Bayley assessment, with children scoring < 85 on either the MDI or PDI classified as having neurodevelopmental delay (ND). This resulted in the following groups: C/TD, control mother (low/no alcohol consumption), typically developing child (*n* = 15); C/ND, control mother, neurodevelopmental delay in the child (*n* = 12); A/TD, alcohol-consuming mother, typically developing child (*n* = 15); and A/ND, alcohol-consuming mother, neurodevelopmental delay in the child (*n* = 17).

### Blood collection

Blood samples were collected during a study-related clinic visit scheduled when the children were 2–3.5 years of age. Blood samples were collected by venipuncture into EDTA-coated tubes, and children were not required to fast prior to sample collection. Samples were centrifuged, and plasma aliquoted and stored at − 80 °C until shipped to the USA where they were stored at − 80 °C until assayed.

### Cytokine assays

Plasma cytokine levels were measured using the Meso Scale Discovery (MSD) V-PLEX Human Biomarker 40-Plex kit (K15209D-1, MSD, Rockville, MD). Plates were read using a MESO QuickPlex SQ120 and data analyzed using the MSD Discovery Workbench software v.4.0. For the lower limit of detection and unabbreviated cytokine list, see Additional file 1: Table S1.

### Statistical modeling

Analysis of maternal and child characteristics was performed using the Kruskal-Wallis rank sum test, the Fisher’s exact test, or the chi-square test (indicated in Tables 1 and 2), with post hoc *p* values adjusted for multiple comparisons using the Holm-Bonferroni method. Cytokine levels below the detection limit of the assay were assigned a value of zero and outliers (> |3.29| standard deviations above/below the mean) were Winsorized. The following cytokines were undetectable in over 10% of the samples and were excluded from the analyses (with the exception of the heatmap)—GM-CSF (27%), IL-1α (49%), IL-4 (58%), IL-13 (73%), IL-1β (73%), and IL-12p70 (92%). Cytokine values were non-normally distributed and were Blom transformed for statistical analysis.

**Table 1:** Maternal characteristics

Variable		C/TD (*n* = 15)	C/ND (*n* = 12)	A/TD (*n* = 15)	A/ND (*n* = 17)	*p* value
Maternal age at enrollment (years)		27.5 ± 1.3	27.8 ± 1.5	27.3 ± 2.2	30.2 ± 1.7	0.758^1^
Recruitment site	Khmelnytsky	67.7% (10)	41.7% (5)	53.3% (8)	82.4% (14)	0.126^2^
	Rivne	33.3% (5)	58.3% (7)	46.7% (7)	17.6% (3)	
Marital status	Married or cohabitating	93.3% (14)	100.0% (12)	93.3% (14)	88.2% (15)	0.899^2^
	Single/separated	6.7% (1)	0.0% (0)	6.7% (1)	11.8% (2)	
Maternal education level	Incomplete high school diploma	0.0% (0)	8.3% (1)	0.0% (0)	17.6% (3)	0.263^2^
	High school diploma/vocational school	40.0% (6)	33.3% (4)	40.0% (6)	58.8% (10)	
	Some college (college degree or unfinished university education)	13.3% (2)	8.3% (1)	26.7% (4)	5.9% (1)	
	University graduate	46.7% (7)	50.0% (6)	33.3% (5)	17.6% (3)	
Socio-economic status category (Hollingshead score)	1 (high), 55–66	33.3% (5)	16.7% (2)	6.7% (1)	0.0% (0)	0.226^2^
	2, 40–54	33.3% (5)	25.0% (3)	26.7% (4)	29.4% (5)	
	3, 30–39	13.3% (2)	25.0% (3)	40.0% (6)	23.5% (4)	
	4, 20–29	12.5% (2)	33.3% (4)	13.3% (2)	17.6% (3)	
	5 (low), 8–19	6.7% (1)	0.0% (0)	13.3% (2)	29.4% (5)	
Gravidity	> 1	46.7% (7)	75.0% (9)	73.3% (11)	64.7% (11)	0.382^2^
	1	53.3% (8)	25.0% (3)	26.7% (4)	35.3% (6)	
Parity	> 0	46.7% (7)	66.7% (8)	60.0% (9)	52.9% (9)	0.742^3^
	0	53.3% (8)	33.3.3% (4)	40.0% (6)	47.1% (8)	
Pre-pregnancy body mass index (BMI)		23.6 ± 1.1	23.3 ± 1.1	23.3 ± 0.9	25.0 ± 1.1	0.648^1^
Smoking status	Current smoker	6.7% (1)	0.0% (0)	33.3% (5)	43.8% (7)	*0.014*^*2*^
	Never smoked	86.7% (13)	91.7% (11)	53.3% (8)	43.8% (7)	
	Quit after realized pregnant	6.7% (1)	0.0% (0)	6.7% (1)	12.5% (2)	
	Quit before pregnancy	0.0% (0)	8.3% (1)	6.7% (1)	0.0% (0)	
Ounces of alcohol/day at time of conception (AAD0)		0.02 ± 0.02	0.01 ± 0.01	0.69 ± 0.19^a,b^	1.91 ± 0.42^a,b,c^	*< 0.001*^*1*^
Ounces of alcohol/drinking day at conception (AADD0)		0.06 ± 0.05	0.10 ± 0.10	1.73 ± 0.41^a,b^	4.03 ± 0.89^a,b^	*< 0.001*^*1*^
Ounces of alcohol/day 2 weeks prior to enrollment (AADXP)		0.00 ± 0.00	0.004 ± 0.00	0.31 ± 0.13^a,b^	0.65 ± 0.23^a,b^	*< 0.001*^*1*^
Ounces of alcohol/drinking day 2 weeks prior to enrollment (AADDXP)		0.00 ± 0.00	0.05 ± 0.05	0.96 ± 0.30^a,b^	1.38 ± 0.47^a,b^	*< 0.001*^*1*^

*Abbreviations*: *C/TD* control mother (low to no alcohol), typically developing child; *C/ND* control mother (low to no alcohol), neurodevelopmental delay in the child; *A/TD* alcohol-consuming mother, typically developing child; *A/ND* alcohol-consuming mother, neurodevelopmental delay in the child. Continuous variables are reported as the average value ± the standard error of the mean (SEM). Categorical variables are reported as percentage of group followed by the *N* in parentheses. Note: One subject in the A/ND group failed to report their smoking status. ^1^Kruskal-Wallis rank sum test. ^2^Fisher’s exact test. ^3^Chi-square test. *p* values in italics are statistically significant; post hoc testing—^a^> C/TD; ^b^> C/ND; ^c^> A/TD

**Table 2:** Child characteristics

Variable		C/TD (*n* = 15)	C/ND (*n* = 12)	A/TD (*n* = 15)	A/ND (*n* = 17)	*p* value
Age at blood draw (years)		3.68 ± 0.20	3.61 ± 0.23	3.14 ± 0.29	3.60 ± 0.21	0.238^1^
Child sex	Male	46.7% (7)	58.3% (7)	53.3% (8)	58.8% (10)	0.902^3^
	Female	53.3% (8)	41.7% (5)	46.7% (7)	41.2% (7)	
Height < 10th percentile	No	100.0% (15)	83.3% (10)	78.6% (11)	56.3% (9)^❋^	*0.019*^*2*^
	Yes	0.0% (0)	16.7% (2)	21.4% (3)	43.8% (7)	
Weight < 10th percentile	No	93.3% (14)	83.3% (10)	78.6% (11)	50.0% (8)	*0.045*^*2*^
	Yes	6.7% (1)	16.7% (2)	21.4% (3)	50.0% (8)	
Occipital-frontal circumference < 10th percentile	No	100.0% (15)	91.7% (11)	85.7% (12)	62.5% (10)	*0.029*^*2*^
	Yes	0.0% (0)	8.3% (1)	14.3% (2)	37.5% (6)	
Smooth philtrum	No	86.7% (13)	66.7% (8)	85.7% (12)	56.3% (9)	0.174^2^
	Yes	13.3% (2)	33.3% (4)	14.3% (2)	43.8% (7)	
Thin vermilion border	No	86.7% (13)	75.0% (9)	92.9% (13)	62.5% (10)	0.267^2^
	Yes	13.3% (2)	25.0% (3)	7.1% (1)	37.5% (6)	
Palpebral fissure length < 10th percentile	No	100.0% (15)	91.7% (11)	92.9% (13)	68.8% (11)	0.052^2^
	Yes	0.0% (0)	8.3% (1)	7.1% (1)	31.3% (5)	
Age at 1st exam (years)		0.56 ± 0.01	0.51 ± 0.02	0.55 ± 0.02	0.56 ± 0.20	0.059^1^
Mental development index (1st exam)		91.67 ± 0.87	82.08 ± 3.40^❋^	91.87 ± 0.98^⦿^	80.87 ± 2.05^❋,✦^	*< 0.001*^*1*^
Psychomotor development index (1st exam)		92.00 ± 2.13	81.92 ± 2.75^❋^	88.40 ± 2.19	80.47 ± 3.28^❋^	*< 0.007*^*1*^
Age at 2nd exam (years)		1.05 ± 0.02	0.99 ± 0.01	1.05 ± 0.02	1.02 ± 0.01	0.084^1^
Mental development index (2nd exam)		94.93 ± 2.77	84.55 ± 2.36^❋^	96.93 ± 2.06^⦿^	76.63 ± 2.06^❋,✦^	*< 0.001*^*1*^
Psychomotor development index (2nd exam)		107.36 ± 3.2	92.45 ± 3.95	102.43 ± 2.60	83.81 ± 3.60^❋,✦^	*< 0.001*^*1*^

*Abbreviations*: *C/TD* control mother (low to no alcohol), typically developing child; *C/ND* control mother (low to no alcohol), neurodevelopmental delay in the child; *A/TD* alcohol-consuming mother, typically developing child; *A/ND* alcohol-consuming mother, neurodevelopmental delay in the child. Continuous variables are reported as average value ± standard error. Categorical variables are reported as percentage of group followed by the *N* in parentheses. For both the A/TD and A/ND groups, one subject did not complete the dysmorphology exam. For the neurodevelopmental assessments, two subjects from the A/ND group did not complete the 6-month assessment and one subject from each group did not complete the 12-month assessment, with no subjects failing to complete the assessment at both time points. ^1^Kruskal-Wallis rank sum test. ^2^Fisher’s exact test. ^3^Chi-square test. *p* values in italics are statistically significant; post hoc testing—^❋^< C/TD; ^⦿^> C/ND; ^✦^< A/TD

To explore the relationship between individual cytokines and group membership (C/TD, C/ND, A/TD, and A/ND), hierarchical linear regression models were fit for each cytokine, with adjustment for maternal smoking, pre-pregnancy body mass index (BMI), child sex, and child age at the time of blood collection. *p* values were considered significant at *p* ≤ 0.05.

In order to visualize the levels of all 40 cytokines and to compare the global cytokine patterns across groups, heatmaps depicting the average profile in each group (average per group; built on *z*-scored data) were generated. Next, as cytokines do not function independently and it is the immune milieu as a whole that impacts outcomes, we took a multivariate approach by utilizing constrained principle component analysis (CPCA), as has been done previously [12]. This method allows for the identification of networks of interacting cytokines and for the determination of the degree of involvement/importance of these networks among groups. CPCA combines multivariate multiple regression and principal component analysis (PCA) into a unified framework, allowing for the identification of networks (components) that are specifically predictable from the independent variables [46, 47]. First, the matrix of dependent variables (cytokines) was regressed to the independent variables (group), resulting in a matrix of predicted scores that reflect the variance in cytokine levels that is predictable based on group membership (predictable variance). Next, a PCA was computed on the predicted scores, which revealed the cytokine networks that were affected by group membership. The PCA solutions were separately rotated using Varimax with Kaiser normalization, and the number of components was extracted based on scree plots [48]. Finally, to determine the degree to which group membership impacted the cytokine networks, correlations were computed between the variable coding groups and the component scores from each of the extracted components.

All statistical analyses were performed using IBM SPSS Statistics for Macintosh (version 25.0, Armonk, NY: IBM Corp), with the exception of the CPCA, which was performed using Matlab (R2014b, Massachusetts, USA). Heatmaps were generated using R statistical software (RStudio Team, 2013, Vienna, Austria, R Foundation for Statistical Computing).

## Results

### Sample characteristics

#### Maternal

There were no group differences in the following maternal characteristics—age at enrollment, marital status, education level, socio-economic status, gravidity, parity, or pre-pregnancy body mass index (Table 1). In addition, there were no group differences in the proportion of subjects recruited from the Khmelnytsky and Rivne prenatal care facilities. By contrast, while smoking status was found to differ among groups (*p* = 0.014), following Holm-Bonferroni correction, post hoc *p* values were all non-significant (Table 1). As expected, all alcohol consumption variables differed by group; post hoc testing showed that alcohol consumption was higher in both alcohol-consuming groups (A/TD, A/ND), compared to the low/no alcohol-consuming groups (C/TD, C/ND). In addition, for one alcohol consumption variable (the number of ounces of alcohol consumed per day at the time of conception [AAD0]), we observed higher levels for alcohol-consuming mothers of children with neurodevelopmental delay (A/ND) compared to their counterparts with typically developing children (A/TD) (Table 1).

#### Child

There were no group differences in the following child characteristics—age at the time of blood draw, sex, and age at the time of neurodevelopmental testing (6 and 12 months; Table 2). In addition, for the dysmorphology data, there were no group differences in the number of children with a smooth philtrum or thin vermilion border, or with palpebral fissure length below the tenth percentile. However, there were group differences in the number of children with height, weight, and occipital-frontal circumference falling below the tenth percentile. Weight and occipital-frontal circumference were no longer significantly different among groups following Holm-Bonferroni correction. For height, however, a greater proportion of children were below the tenth percentile in the A/ND group, as compared to the C/TD group (Table 2). As groups were defined, in part, based on childhood neurodevelopmental outcome (typical neurodevelopment or neurodevelopmental delay), group differences were detected for both the MDI and PDI exam scores. Generally, as expected, MDI and PDI scores were lower for children with neurodevelopmental delay as compared to their typically developing counterparts. Of note, the MDI scores were more consistently lower in the neurodevelopmental delay groups: the MDI scores at 6 and 12 months were lower for both neurodevelopmental delay groups (C/ND and A/ND) compared to both typically developing group (C/TD and A/TD), with more subtle differences detected for the PDI scores (Table 2).

### Analysis of individual cytokine levels

Following adjustment for covariates (maternal smoking, pre-pregnancy BMI, child sex, and child age at the time of blood collection), linear regression analyses identified a differential cytokine pattern for bFGF (*R*^2^ = 0.31, *p* = 0.033) only, with higher bFGF in the A/ND compared to the C/ND group.

### Global cytokine profiles

**Fig. 1:**
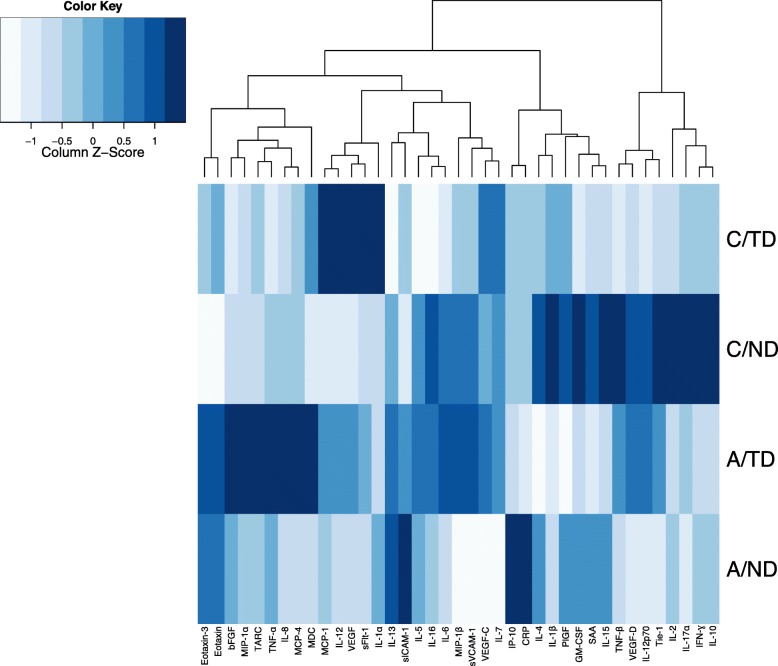
Heatmaps showing the overall cytokine profile. Rows represent groups (C/TD, C/ND, A/TD, A/ND), as indicated, and columns represent mean cytokine levels (*z*-scored data), for each group. Colors demonstrate deviations from the mean of zero, as indicated in the color key. Abbreviations: C/TD, control mother (low to no alcohol), typically developing child (*n* = 15); C/ND, control mother (low to no alcohol), neurodevelopmental delay in the child (*n* = 12); A/TD, alcohol-consuming mother, typically developing child (*n* = 15); A/ND: alcohol-consuming mother, neurodevelopmental delay in the child (*n* = 17)

Examination of global cytokine profiles (Fig. 1) revealed differential overall patterns based on group membership. Importantly, for both controls and alcohol-exposed children, differential cytokine patterns were detected based on neurodevelopmental outcome. For controls, we found higher average levels of a subset of cytokines including IL-4, IL-1β, PlGF, GM-CSF, SAA, IL-15, TNF-β, VEGF-D, IL-12p70, Tie-2, IL-2, IL-17α, IFN-ɣ, and IL-10 in children with neurodevelopmental delay (C/ND), compared to that of their typically developing (C/TD) counterparts (Fig. 1). Similarly, differences in the overall cytokine profiles between the two alcohol-exposed groups indicated higher mean levels of eotaxin-3, eotaxin, bFGF, MIP-1α, TARC, TNF-α, IL-8, MCP-4, and MDC detected in the A/TD group compared to the A/ND group (Fig. 1). Finally, for children with neurodevelopmental delay, the overall cytokine profile differed between those with alcohol-independent (C/ND) vs. alcohol-dependent (A/ND) delay. Specifically, the subset of cytokines detected at higher mean levels in the control condition (C/ND)—IL-4, IL-1β, PlGF, GM-CSF, SAA, IL-15, TNF-β, VEGF-D, IL-12p70, Tie-2, IL-2, IL-17α, IFN-ɣ, IL-10—was generally dampened with alcohol-exposure (A/ND).

### Cytokine network analysis

**Fig. 2:**
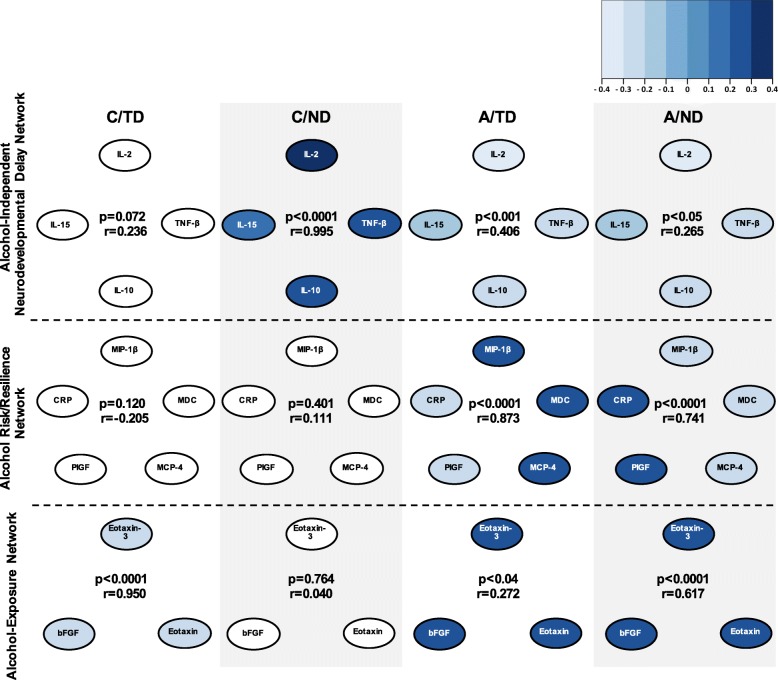
Cytokines contributing to the three immune networks identified through constrained principle component analysis (CPCA). Network membership was defined based on component loadings from the CPCA. Activated cytokines are indicated in dark blue, and inhibited cytokines indicated in light blue, with color gradation depicting the value of the component loading. For each network, the strength (|r|) and the significance (*p* value) of the correlation between groups (C/TD, C/ND, A/TD, A/ND) and component scores are indicated. The cytokine networks indicated in white were not significantly correlated with the group, as indicated in the figure. Abbreviations: C/TD, control mother (low to no alcohol), typically developing child (*n* = 15); C/ND, control mother (low to no alcohol), neurodevelopmental delay in the child (*n* = 12); A/TD, alcohol-consuming mother, typically developing child (*n* = 15); A/ND, alcohol-consuming mother, neurodevelopmental delay in the child (*n* = 17)

To take a multivariate approach extending beyond individual cytokine profiles to probe for group differences in the overall cytokine milieu, a CPCA was conducted. The CPCA revealed a three-network (or component; Additional file 1: Table S2) solution; Fig. 2 summarizes the cytokines that were activated (dark blue) or inhibited (light blue) in each network. In addition, to illustrate differential network involvement among groups, the strength (|r|) and significance (*p* value) of the correlation for each group are noted.

Network 1 included IL-2, TNF-β, IL-10, and IL-15 and was defined as the *Alcohol-Independent Delay Network*, as this network was activated specifically in controls with neurodevelopmental delay. Network 2 included MIP-1β, MDC, MCP-4, PlGF, and CRP and was defined as the *Alcohol-Associated Risk* vs. *Resilience Network*, as cytokines belonging to this network showed a differential pattern between the two alcohol-exposed groups. Alcohol-exposed children with typical neurodevelopment (A/TD; resilient) showed activation of MIP-1β, MDC, and MCP-4 and inhibition of CRP and PlGF, with the opposing profile detected in alcohol-exposed children with neurodevelopmental delay (A/ND; at risk). Finally, network 3 included eotaxin-3, eotaxin, and bFGF and was defined as the *Alcohol-Exposure Network*, as this network was activated in both alcohol-exposed groups (A/TD and A/ND) and inhibited in control children with typical neurodevelopment (C/TD). Of note, while both alcohol-exposed groups show the same pattern for network 1 vs. network 3 activity (inhibition of network 1 and activation of network 3), the differential pattern of activity detected for network 2 allowed for the dissociation between the cytokine profile of alcohol-exposed children with (A/ND) and without (A/TD) neurodevelopmental delay. Finally, for both control groups, a single network was found to be involved. Specifically, for control children with neurodevelopmental delay (C/ND), network 1 was activated, and for control children with typical neurodevelopment (C/TD), network 3 was inhibited (Fig. 2).

## Discussion

In the current study, we identify unique immune profiles associated with prenatal exposure to alcohol, as well as differential immune profiles linked to neurodevelopmental status (typical development vs. neurodevelopmental delay). Importantly, cytokines do not function independently but rather as components in complex immune networks, together orchestrating the development and function of a range of neural and physiological systems (reviewed in [38, 39]), yet despite this, cytokines are most commonly evaluated independently in the literature to date. To better account for the complexity of immune function and in order to visualize overall patterns of all cytokines measured, two multivariate techniques were employed in the current study—heatmaps, for data visualizing, and CPCA, for statistical network analysis. The graphical representation of cytokine levels in the heatmap allowed the immune milieu to be collectively compared between groups and demonstrated visually that the cytokine profile differed based on maternal alcohol consumption and child neurodevelopmental status. Indeed, inspection of the heatmaps indicates that the immune milieu is distinct in controls with neurodevelopmental delay (C/ND), compared to their typically developing counterparts (C/TD). The distinction between immune profiles is also apparent in the comparison between children experiencing alcohol-independent (C/ND) compared to alcohol-dependent (A/ND) neurodevelopmental delay. Building on these differential cytokine profiles, levels of 33 cytokines were analyzed simultaneously through the data reduction approach of CPCA, allowing for the identification of cytokine networks showing differential activity patterns between groups of children based on alcohol exposure and neurodevelopmental status. A specific advantage of this network approach, beyond being a better representation of cytokine signaling than measures of individual cytokines, is that the variability in individual cytokines can be combined such that subtle but potentially important patterns can be identified. For example, low-grade systemic inflammation is implicated in a range of adult conditions and diseases [49–52], and as a result, identification of subclinical inflammatory profiles before they reach the threshold of overt inflammation may be especially important in predicting and preventing disease. Moreover, early detection of this low-grade inflammation may only be possible when groups of cytokines are examined together.

Through this network approach, the current study explored whether cytokine profiles during childhood could be informative in identifying whether children were prenatally exposed to alcohol as well as in predicting the risk of vs. resilience to subsequent neurodevelopmental disorders. Our novel approach demonstrated that unique cytokine networks can be associated with both alcohol exposure and neurodevelopmental status. Control children experiencing neurodevelopmental delay (C/ND) showed activation of what we defined as the Alcohol-Independent Delay Network (network 1) that included IL-2, TNF-β, IL-10, and IL-15. Notably, this network was not activated in any other group. By contrast, prenatal alcohol exposure, regardless of neurodevelopmental status, resulted in activation of the Alcohol-Exposure Network (network 3) that included eotaxin-3, eotaxin, bFGF but inhibition of the Alcohol-Independent Delay Network (network 1). Furthermore, involvement of the Alcohol-Associated Risk vs. Resilience Network (network 2) differed based on neurodevelopmental outcome—alcohol-exposed children who were typically developing (A/TD) showed activation of MIP-1β, MDC, and MCP-4, and inhibition of CRP and PlGF, with the opposing pattern of cytokine activation/inhibition detected in alcohol-exposed children with neurodevelopmental delay (A/ND), suggesting that activity of this network may play a role in child neurodevelopmental risk vs. resilience. Importantly, the overall patterns identified in the network analysis parallel the global cytokine patterns displayed in the heatmap. Finally, the finding of differential cytokine profiles for alcohol-dependent and alcohol-independent neurodevelopmental delay is significant, as it shows for the first time that we can identify an insult-specific immune milieu and distinguish it from an immune milieu associated with neurodevelopmental delay in controls who have no known prenatal insult.

Children with alcohol-independent neurodevelopmental delay (C/ND) showed activation of a single network, the Alcohol-Independent Neurodevelopmental Delay Network (network 1; IL-2, IL-15, TNF-β, and IL-10). Previous studies provide evidence for changes in this particular subset of cytokines in other neurodevelopmental disorders. Elevated IL-2 (serum [54];) and TNF-β (amniotic fluid [53];) have both been previously associated with ASD. In addition, while elevated levels of IL-10 have not typically been reported in children with ASD [31], IL-10 levels may be elevated in children with more severe ASD [9, 55]. Of note, however, there are well-acknowledged inconsistencies in the cytokine profiles reported with ASD, likely due to a multitude of factors including methodological differences in cytokine profiling techniques, differences in key experimental features between studies, and the overall heterogeneous nature of ASD (reviewed in [34]). Finally, while IL-15 is not a commonly measured cytokine in studies of neurodevelopmental disorders, there is evidence for elevated levels of IL-15 in Rett syndrome [56]. Based on our findings, we propose that elevations in IL-2, IL-15, TNF-β, and IL-10 may represent a non-specific immune profile broadly associated with neurodevelopmental delay during childhood, rather than serving as a more specific biomarker of any one neurodevelopmental disorder. By contrast, for alcohol-exposed children experiencing neurodevelopmental delay (A/ND), network 1 was inhibited, suggesting that activity of this cytokine network may be, at least in part, insult-specific.

It has been shown previously that alcohol consumption during pregnancy impacts maternal cytokine levels [12, 30], with maternal cytokine profiles also differing based on child neurodevelopmental status (alcohol-dependent neurodevelopmental delay or typical neurodevelopment) [12]. Moreover, while data from animal models has linked prenatal alcohol exposure with cytokine disturbances in the offspring [57], the present data are the first to show an association between alcohol exposure and long-lasting alterations in cytokine levels in children. Here, irrespective of neurodevelopmental outcome, prenatal alcohol exposure resulted in activation of eotaxin-3, eotaxin, and bFGF, which we defined as the Alcohol-Exposure Network (network 3). Broadly, these immunomodulators have all been associated with the pathogenesis of asthma. Eotaxin and exotaxin-3 are potent modulators of eosinophils, promoting eosinophil recruitment to the lung [58, 59]. Elevated levels of eotaxin, eotaxin-3, and bFGF have also been detected in the plasma [60], airway epithelium [61], and bronchial submucosa [62], respectively, of asthmatic patients. Of relevance, while asthma-related outcomes have not been extensively investigated in children prenatally exposed to alcohol, there are reports of subtle trends for increased asthma rates in children [26] and adolescents [63] with prenatal alcohol exposure, as well as data from animal models showing impairments in the immune environment of the lung [64–66]. As such, in the context of prenatal alcohol exposure, activation of this immune network could be playing a role in early asthma pathophysiology, as well in other related eosinophilic conditions, including atopic dermatitis, which also occurs at higher rates following prenatal alcohol exposure [25, 67].

In addition to exploring whether prenatal alcohol exposure has a unique impact on cytokine levels in childhood, a key objective of the current study was to determine whether the cytokine profiles of alcohol-exposed children differed based on their neurodevelopmental status. This is particularly important due to the fact that there is well-established heterogeneity in the effects of prenatal alcohol exposure that is likely related to a multitude of factors including timing, pattern, and amount of alcohol exposure; socioeconomic status; drug use; and genetics [68]. Identification of a specific immune signature associated with alcohol-related neurodevelopmental delay could aid in the early identification of especially vulnerable children with the goal of implementing early intervention strategies. In the current study, the immune profile of these at-risk children (A/ND) could be disentangled from that of their neurotypical counterparts (A/TD) by analyzing the activity of Alcohol-Associated Risk vs. Resilience Network (network 2): CRP and PlGF levels were elevated and MIP-1β, MDC, and MCP-4 were inhibited in alcohol-exposed children with neurodevelopmental delay, with the opposing profile detected in children with typical neurodevelopment. Thus, the activity of network 2 may be particularly important in predicting child risk vs. resilience, with elevated levels of MIP-1β, MDC, and MCP-4 potentially acting as protective factors, whereas elevated levels of PlGF and CRP potentially acting as risk factors for neurodevelopmental delay.

Both PlGF and CRP have been implicated in the pathophysiology of numerous diseases. PlGF is an essential angiogenic factor for placental development and postnatally stimulates the atherogenic migration of macrophages into the arterial wall [69], playing a key role in early atherosclerosis and cardiovascular disease [70, 71]. Similarly, persistent elevations in CRP, or low-grade inflammation, have been linked to higher risk of coronary heart disease [72] and in the pathophysiology of atherosclerosis (reviewed in [73]). While additional research is needed to understand the impact of prenatal alcohol exposure on the cardiovascular system [74, 75], FASD has been associated with higher rates of congenital heart defects [76] and studies using animal models have shown adverse cardiovascular effects in adulthood such as elevated systolic blood pressure [77] and left ventricular hypertrophy [78] following prenatal alcohol exposure. In addition, childhood adversity/maltreatment [79, 80] and adult depression [81], both alone and in combination [82], are also associated with elevations in CRP. As individuals prenatally exposed to alcohol are at higher risk of experiencing adverse environments during early postnatal life [83] and mental health problems including depression [84], this association and involvement of CRP merits further investigation in longitudinal studies. Support for this also comes from rodent studies showing that prenatal alcohol exposure alone or in combination with early-life adversity is also associated with elevated CRP levels in the early postnatal period [85]. Taken together, the activation of PlGF and CRP in at-risk children (A/ND) in the current study is intriguing and warrants further follow-up to understand whether alterations in the activity of cytokine networks, beyond being associated with neurobehavioral phenotype, may also be suggestive of low-grade inflammation that may be predictive of vulnerability to later life diseases/disorders such as cardiovascular disease and mental health problems. This is particularly important as the field of FASD research expands from a “brain-centric” to a “whole-body disorder” approach.

Finally, while the current study identified cytokine profiles associated with childhood neurodevelopmental risk and resilience, both in the context of prenatal alcohol exposure and with low/no exposure, possible limitations should also be considered. First, while self-reports of alcohol consumption can provide reliable and valid information, there are known limitations/issues, such as reporting shown to be influenced by factors including social context, respondent characteristics, and test attributes [86]. While many of these factors were considered in the current study design, it stands to reason that self-report of alcohol consumption may not always accurately capture drinking behaviors. Secondly, while the BSID has been viewed as the “gold standard” tool for assessment as well as research involving both infants and children [87], there are known limitations to this assessment tool as well, including a lack of research examining the stability of this test across repeat assessments [88]. For example, while the MDI is the most commonly used measure to assess cognitive function in at-risk infants, the predictive validity of a low MDI score has been found to be low in certain high-risk groups [89, 90]. In addition, while the BSID is considered a valid and important indicator of current functioning, in certain high-risk groups, mental development is known to decelerate or change across development [90]. As a result, in order to ascertain whether cytokine profiles associated with infant neurodevelopmental delay as indicated by BSID scores are predictive of long-term neurodevelopmental delay in both alcohol-exposed and control infants, evaluation of neurodevelopment at later stages during childhood is required. Finally, it is important to note that additional validation will be required in order to determine whether the described cytokine signatures are consistent in different populations (e.g., in North America), across different time points (e.g., at later points during childhood), and in larger samples. Despite the relatively small sample size of the current study, the data provide compelling evidence supporting the inclusion of a wide array of cytokines/chemokines and related factors in studies evaluating the relationship between inflammation and childhood risk/resilience. In addition, the current dataset also supports the use of statistical techniques aimed at evaluating groups of cytokines, or cytokine networks, in order to better represent the complex inflammatory milieu.

## Conclusions

In conclusion, through a novel and promising network approach, distinct immune profiles were identified that could distinguish whether the child had been exposed to alcohol, as well as whether or not they showed neurodevelopmental delay. Together, these data suggest that despite some overlap in neurobehavioral profiles, prenatal alcohol exposure has a unique impact on the childhood immune milieu. In addition, differential immune profiles were identified in children prenatally exposed to alcohol who showed developmental delay (A/ND; more at risk), compared to those who were typically developing (A/TD; more resilient). While there have been extensive investigations into the cytokine disturbances associated with other neurodevelopmental disorders such as ASD, this is the first study to evaluate the cytokine profile in children with prenatal alcohol exposure. Continued research efforts are required to further characterize the immune profile across the life course and to identify the mechanisms by which immune changes may act to modulate neurodevelopment as well as other whole-body health outcomes. Finally, in the context of neurodevelopment, the identification of immune signatures associated with risk vs. resilience, for both unexposed and alcohol-exposed children, is highly significant, providing a first step in the search for biomarkers that would allow for early identification of at-risk children who could then be targeted for early intervention.

## Supplementary information

**Additional file 1 Table S1**. Cytokine Assay Lower Limits of Detection. **Table S2**. Component Loadings.

## Data Availability

The data used in this study are available from the corresponding author upon reasonable request and CIFASD approval.

## Abbreviations

A/ND: Alcohol-consuming mother, neurodevelopmental delay in the child
A/TD: Alcohol-consuming mother, typically developing child
ASD: Autism spectrum disorder
bFGF: Basic fibroblast growth factor
BMI: Body mass index
BSID-II: Bayley Scales of Infant Development second edition
C/ND: Control mother, neurodevelopmental delay in the child
C/TD: Control mother (low/no alcohol consumption), typically development child
CIFASD: Collaborative Initiative on Fetal Alcohol Spectrum Disorders
CP: Cerebral palsy
CPCA: Constrained principle component analysis
CRP: C-reactive protein
FAS: Fetal alcohol syndrome
FASD: Fetal alcohol spectrum disorders
GM-CSF: Granulocyte-macrophage colony stimulating factor
IL-10: Interleukin-10
IL-12p70: Interleukin-12p70
IL-13: Interleukin-13
IL-15: Interleukin-15
IL-1α: Interleukin-1α
IL-1β: Interleukin-1β
IL-2: Interleukin-2
IL-4: Interleukin-4
IL-6: Interleukin-6
IL-8: Interleukin-8
MCP-4: Monocyte chemotactic protein 4
MDC: Macrophage-derived chemokine
MDI: Mental Development Index
MSD: Meso Scale Discovery
PCA: Principle component analysis
PDI: Psychomotor Development Index
PlGF: Placental growth factor
SAA: Serum amyloid A
TARC: Thymus and activation regulated chemokine
Tie-2: Tyrosine kinase-2
TNF- β: Tumor necrosis factor β
TNF-α: Tumor necrosis factor α
VEGF-D: Vascular endothelial growth factor-D
