# Supplementary material for: Immune network dysregulation associated with child neurodevelopmental delay: modulatory role of prenatal alcohol exposure
Source: J Neuroinflammation. 2020 Jan 28;17:39. doi: 10.1186/s12974-020-1717-8 (PMC6988366; doi:10.1186/s12974-020-1717-8)
Supplement: Supplementary file 1 — Additional file 1 Table S1. Cytokine Assay Lower Limits of Detection. Table S2. Component Loadings. [file 12974_2020_1717_MOESM1_ESM.docx]

**Table S1.** Cytokine Assay Lower Limits of Detection

| **Cytokine** | | **LLOD (pg/mL)** |
| --- | --- | --- |
| C-reactive protein | CRP | 3.21 – 3.93 |
| Eotaxin | Eotaxin  (or CCL11) | 3.58 – 3.77 |
| Eotaxin-3 | Eotaxin-3  (or CCL26 or MIP-4α) | 0.61 – 1.63 |
| Fms-like tyrosine kinase/vascular endothelial growth factor receptor 1 | sFlt-1/VEGFR1 | 0.68 – 0.77 |
| Basic fibroblast growth factor | bFGF | 0.08 – 0.09 |
| Granulocyte-macrophage colony stimulating factor | GM-CSF | 0.06 – 0.15 |
| Intercellular adhesion molecule-1 | sICAM-1 | 1.68 – 1.87 |
| Interferon-γ | IFN-γ | 0.16 – 0.43 |
| Interleukin-1α | IL-1α | 0.25 – 0.26 |
| Interleukin-1β | IL-1β | 0.04 – 0.07 |
| Interleukin-10 | IL-10 | 0.02 – 0.04 |
| Interleukin-12p70 | IL-12p70 | 0.05 – 0.25 |
| Interleukin-12/Interleukin-23 p40 | IL-12/IL-23p40 | 0.23 – 0.26 |
| Interleukin-13 | IL-13 | 0.21 – 0.35 |
| Interleukin-15 | IL-15 | 0.06 – 0.08 |
| Interleukin-16 | IL-16 | 0.32 – 0.59 |
| Interleukin-17A | IL-17A | 0.13 – 0.30 |
| Interleukin-2 | IL-2 | 0.02 – 0.06 |
| Interleukin-4 | IL-4 | 0.01 – 0.02 |
| Interleukin-5 | IL-5 | 0.24 – 0.25 |
| Interleukin-6 | IL-6 | 0.04 |
| Interleukin-7 | IL-7 | 0.06 – 0.13 |
| Interleukin-8 | IL-8  (or CXCL8) | 0.03 – 0.05 |
| Interferon gamma-induced protein | IP-10  (or CXCL10) | 0.07 – 0.42 |
| Monocyte chemotactic protein 1 | MCP-1  (or CCL2) | 0.04 – 0.25 |
| Monocyte chemotactic protein 4 | MCP-4  (or CCL13) | 3.38 – 4.78 |
| Macrophage-derived chemokine | MDC  (or CCL22) | 2.72 – 21.3 |
| Macrophage inflammatory protein 1α | MIP-1α  (or CCL3) | 3.07 – 7.52 |
| Macrophage inflammatory protein 1β | MIP-1β  (or CCL4) | 0.48 – 2.68 |
| Placental growth factor | PlGF | 0.30 – 0.51 |
| Serum amyloid A | SAA | 14.4 – 16.1 |
| Thymus and activation regulated chemokine | TARC  (or CCL17) | 0.07 – 0.23 |
| Tyrosine kinase-2 | Tie-2 | 32.40 – 34.80 |
| Tumor necrosis factor α | TNF-α | 0.04 – 0.07 |
| Tumor necrosis factor β | TNF-β | 0.04 – 0.06 |
| Vascular cell adhesion molecule-1 | sVCAM-1 | 9.11 – 10.10 |
| Vascular endothelial growth factor-A | VEGF-A | 0.14 – 0.46 |
| Vascular endothelial growth factor-C | VEGF-C | 8.68 – 8.80 |
| Vascular endothelial growth factor-D | VEGF-D | 2.52 – 4.51 |

The lower limit of detection (LLOD) varied by plate and analyte. LLOD ranges are presented in the table.

**Table S2.** Component Loadings

| **Variable** | **Comp 1** | **Comp 2** | **Comp 3** |
| --- | --- | --- | --- |
| IL-2 | **-0.3975** | -0.0703 | -0.1957 |
| IL-10 | **-0.2508** | -0.0564 | -0.1999 |
| TNF-β | **-0.2774** | 0.1307 | 0.0078 |
| IL-15 | **-0.1996** | -0.0504 | -0.1025 |
| MIP-1β | -0.0729 | **0.2196** | -0.0841 |
| PIGF | -0.0557 | **-0.2254** | -0.0795 |
| MDC | 0.1216 | **0.2109** | 0.0665 |
| MCP-4 | -0.0214 | **0.2013** | 0.0631 |
| CRP | -0.0028 | **-0.2032** | 0.0473 |
| Eotaxin-3 | 0.1054 | 0.0717 | **0.2871** |
| Eotaxin | 0.1523 | 0.0620 | **0.2184** |
| bFGF | -0.0300 | 0.0970 | **0.2115** |
| IL-12p40 | 0.0064 | -0.0729 | 0.1687 |
| sICAM-1 | 0.0148 | -0.0170 | 0.0484 |
| sVCAM-1 | -0.0249 | 0.1057 | -0.0027 |
| sFlt-1 | 0.1659 | 0.0856 | -0.1038 |
| IL-16 | -0.1180 | 0.0138 | 0.0551 |
| Tie-2 | -0.2281 | 0.1648 | -0.0716 |
| VEGF | 0.1483 | 0.0870 | -0.0732 |
| VEGFC | 0.0333 | 0.1547 | -0.0730 |
| VEGFD | -0.1697 | 0.1665 | -0.0351 |
| IL-6 | -0.1036 | 0.0680 | 0.0090 |
| IL-8 | -0.0684 | 0.1369 | 0.0397 |
| TNF-α | -0.0299 | 0.0476 | 0.0841 |
| IL-7 | 0.0097 | 0.0515 | -0.1180 |
| TARC | 0.0167 | 0.1543 | 0.0415 |
| IFN-ɣ | -0.1153 | -0.0879 | -0.0987 |
| IL-17α | -0.1747 | 0.0934 | -0.1541 |
| MCP-1 | 0.1741 | 0.0392 | -0.0084 |
| IP-10 | -0.0407 | -0.1849 | 0.0372 |
| SAA | -0.0195 | -0.1766 | 0.0251 |
| IL-5 | -0.0190 | -0.0391 | 0.1888 |
| MIP-1α | -0.0148 | 0.1501 | 0.1716 |

Component loadings for the variance in cytokine measures constrained to that predictable from group membership (C/TD, C/ND, A/TD, A/ND). Networks are interpreted using loadings set in **bold**. Shaded cells indicate cytokine measures that were not associated with any component.

Abbreviation: Comp: Component/Network
